# Secondary psychosis induced by metabolic disorders

**DOI:** 10.3389/fnins.2015.00177

**Published:** 2015-05-19

**Authors:** Olivier Bonnot, Paula M. Herrera, Sylvie Tordjman, Mark Walterfang

**Affiliations:** ^1^Psychology Laboratory of Pays de la Loire (LPPL), U2PEA Department of Child and Adolescent Psychiatry, Centre Hospitalier Universitaire de Nantes, University of AngersNantes, France; ^2^Grupo de Investigación en Neurociencias NeURos, Universidad del RosarioBogota, Colombia; ^3^Department of Child an Adolescent Psychiatry, Centre Hospitalier Guillaume Regnier, University of RennesRennes, France; ^4^Neuropsychiatry Unit, Royal Melbourne HospitalMelbourne, VIC, Australia; ^5^Melbourne Neuropsychiatry Centre, University of MelbourneMelbourne, VIC, Australia

**Keywords:** IEMs, neurometabolic disorders, homocysteine, urea, organic psychosis, schizophrenia-like, atypicalness

## Abstract

Metabolic disorders are not well-recognized by psychiatrists as a possible source of secondary psychoses. Inborn errors of metabolism (IEMs) are not frequent. Although their prompt diagnosis may lead to suitable treatments. IEMs are well-known to pediatricians, in particular for their most serious forms, having an early expression most of the time. Recent years discoveries have unveiled later expression forms, and sometimes very discreet first physical signs. There is a growing body of evidence that supports the hypothesis that IEMs can manifest as atypical psychiatric symptoms, even in the absence of clear neurological symptoms. In the present review, we propose a detailed overview at schizophrenia-like and autism-like symptoms that can lead practitioners to bear in mind an IEM. Other psychiatric manifestations are also found, as behavioral, cognitive, learning, and mood disorders. However, they are less frequent. Ensuring an accurate IEM diagnosis, in front of these psychiatric symptoms should be a priority, in order to grant suitable and valuable treatment for these pathologies.

## Introduction

The Neurometabolic Diseases (NMDs), also known as Inborn Errors of Metabolism (IEMs), are genetic disorders affecting, for most of them, the conversion of a substrate into a product by an enzyme.

An insufficient enzyme production can cause the accumulation of the substrate and the absence or depletion of the final expected product. The mechanisms and metabolic processes are obviously much more complex than this simple description. Some pathologies in particular, do not fully meet that definition. Although, this simplified representation may warrant a reasonable explanation to the extreme diversity of organ damages found in the NMDs. Indeed, the absence of an essential product from birth, or the accumulation of deleterious product may have a massive impact on the brain. This pathological situation not only can explain the frequency of neurological or psychiatric signs, but also the presence of anomalies found in other organs, or vice versa. Altered intracellular concentrations is a common physiopathological core on several NMDs, derive from an altered synthesis or a modified catabolism.

Main interest for psychiatrist is the fact that some diseases may emerge with psychiatric signs with no other associated symptoms. Neurological soft signs can appear later, after many years of active psychotic presentation.

Once the metabolic disease is recognized, it is possible to adopt simple therapeutic strategies. For some conditions, this may imply dietary changes in order to avoid non-degradable substrate accumulation, or increasing the intake of an absent product, sometimes a humble vitamin.

Moreover, advances are made in enzyme therapy. The simplicity of the therapeutic plan should be a call to all practitioner for acquiring a better knowledge, and thus recognition, of these treatable diseases.

In this paper, we will introduce some of the NMDs having psychiatric clinical presentation and a potential improvement when receiving suitable treatment. We are going to focus on NDMs leading to schizophrenia like symptoms and some developmental disorders.

## Methods

Data sources has been explored on published literature (MEDLINE sources) inspecting Disorders of Homocysteine Metabolism (DHMs): cystathionine beta-synthase deficiency [CbS-D] and methyltetrahydrofolate reductase deficiency [MTHFR-D], acute porphyria (POR), urea cycle disorders (UCD), cerebrotendinous-xanthomatosis (CTX), Wilson disease (WD), and Niemann-Pick disease type C (NP-C).

Literature scrutiny included all case reports, case series and reviews accounting for original data reporting psychiatric symptoms and cognitive impairments, between a data range from January 1967 and June 2014. A four-step standardized strategy was applied for the selection process.

Selected items were screened for the presence of psychiatric signs and symptoms as key features of disease, natural history, severity and treatments.

A final selection over 619 documents was retained. Documents analyses were conducted as follow: CbS-D (*n* = 6), MRHFR-D (*n* = 4), UCD (*n* = 8), POR (*n* = 12), CTX (*n* = 16), NP-C (*n* = 9). Inclusion was also made for six non-systematic literature published reviews. A lack of explicit description of psychiatric symptoms was observed over several published reports.

All relevant findings will be presented in a didactic perspective, through key features for each disease, the presence of psychiatric symptoms and particular sings suggesting a possible IEM leading to secondary psychosis or developmental events.

## Neurometabolic disease and schizophrenia spectrum disorder

Schizophrenia presents an estimated prevalence of 1% of the general population (Barbato, 1998). Nowadays, there is a widespread knowledge about the fact that patients suffering from schizophrenia have enlarged risks of somatic medical conditions. Complex metabolic syndromes, accounting for hyperglycaemia, dyslipidemia, along with higher global cardiovascular risk for acute myocardial infarction, are listed among the many organic conditions associated with schizophrenic disorders (Price and Ron, 2003). A non-negligent part of these metabolic pathologies can be induced by antipsychotic treatments, as hyperprolactinemia and overweight, but others, as cerebro vascular accidents, are suspected to be facilitated by endothelium specific diseases, not fully understood to date (Harris et al., 2008). Given the extreme complexity of comorbid interactions, it may become difficult to know the exact proportion of these metabolic disorders exhibited by patients in all schizophrenia cases.

The literature review mentions a study conducted on 268 patients, affording 6% prevalence for all organic causes potentially linked to psychotic symptoms (Johnstone et al., 1987). NMDs are accounted among all pathologies, although they are considered as rare diseases, probably under estimated at 4/10,000 in the general population (Applegarth et al., 2000). However, some of them are treatable, which allow us to highlight the interest to know how to make an accurate diagnosis (Bonnot et al., 2014) (Table 1).

**Table 1:** **List of seven NMDs organic and psychiatric symptoms**.

**Core clinical features, diagnosis and treatment hints**					
**Disorder**	**Organic symptoms**	**Psychiatric symptoms**	**Trigger event**	**Biomarkers**	**Treatment**
Homocysteinemia	Thrombolembolism	Intellectual disabilities	Protein diet	Homocysteinimia	
[CbS]	Scoliosis	Mood disorders	Post-surgery	Methioninemia	
	Marfan-like habitus	OCD			
	Cerebellar signs	**Schizophrenia-like sx**			
Homocysteinemia	Apnea^*^	Intellectual Disabilities	High (Met) diet	Homocysteinimia	Vitamin B12
[MTHFR]	Seizures^*^	Visual hallucinations		Methioninemia	Diet^**^
	Microcephaly	**Schizophrenia-like sx**			Pyroxine
	Ataxia				
Urea cycle	Abdominal pain	Mood disorders	Protein diet	Hyperammoniemia	Diet^**^
disorders [UCDs]	Confusion	Intense hallucinations	Post-surgery		
	Nausea/vomiting		Drugs^***^		
Porphyiria	Black/red urine	**Schizophrenia-like sx**	Periodic outburst	PBG in urine	IV hemin
[POR]	Abdominal pain	Delusions		ALA in blood	IV carbohydrate
	Constipation	Hallucinations			
	Nausea/vomiting	Course of though dist.			
	Hepatic disturb.	**Catatonia**			
	Erythropoietic disturb.	Mood disorders			
	Confusion				
Wilson disease	Tremor	Mood disorders		Ceruloplasmin	copper
[WD]	Dystonia	Personality changes			chelation
	Dysarthria	**Schizophrenia-like sx**			
		Hallucinations +++			
	**Kayser-Fleischer ring**	Altered cognitif Fx			
Niemann-Pick	**SNP +++**	**Autism-like sx**	Incidious	Skyn biopsy	Myglustat
disease type C	Deafness^*^	**Schizophrenia-like sx**		Fibroblast culture	
[NP-C]	Neonatal jaundice^*^	Visual hallucinations		Filipin staining	
	Dystonia	Frontal syndrome		[NPC1 and NPC2 gene test]	
	Ataxia				
	Dysartria				
	Dysphagia				
	Splenomegaly				
Cerebrotendinous	Xanthomas	**ADHD**		MRI signs	chenodeoxycholic
xanthomatosis	Chronic diarrhea	**Schizophrenia-like sx**		High cholestanol	Acid
[CTX]	Spastic paralysis	Mood disorders		[CYP27A1 gene test]	
	Epilepsie^*^	Intellectual disabilities			
	Ataxia	Cognitive decline			
	Polyneuropathy				
	Juvenile cataract				

*NMDs, Neurometabolic disorders; OCD, Obsessive compulsive disordes; Met, Methionine; SNP, Supranuclear Palsy; ADHD, Attention Deficit Hyperactive Disorder; MRI, Magnetic Resonance Imaging; sx, symptoms; dist, disturbances. Bold values correspond to noticeable organic or psychiatric symptoms*.

* *Early symptoms after birth or childhood*.

** *Protein restriction diet*,

*** *Trigger drugs reported are corticosteroids and valproic acid*.

## Disorders of homocysteine metabolism (DHMs)

Two pathologies linked to the absence of two distinct enzymes fall into this group: Cystathionine beta synthase deficiency (CbS) and methyltetrahydrofolate reductase deficiency (MTHFR).

*Hyperhomocystinuria due to CbS deficiency* has a prevalence estimated at 1/350,000 births, being an autosomal recessive condition. CbS usually converts homocysteine to cystathionine with the help of three cofactors including vitamin B12 and folic acid. The accumulation of homocysteine is responsible for highly variable clinical features including skeletal abnormalities as a knock-knees or pes cavus, sometimes associated with scoliosis and osteoporosis. One of the morphotypes is a Marfan-like habitus (large size and thinness). Thromboembolic disorders of small or great vessels were presented in 25% of a sample of 15 patients. There are also eye disorders, as lenticular ectopia (in 85% of cases) and severe myopia. Intellectual Disability (ID) is common but not constant.

These signs are frequently associated with psychiatric mood disorders and anxiety disorders. Obsessive compulsive disorder has been reported on half of this population (Abbott et al., 1987), Schizophrenic-like disorders are also possible and can be an isolated clinical feature (Li and Stewart, 1999).

*Homocystinuria related to MTHFR deficiency* is an autosomal recessive defect caused by a mutation in the MTHFR gene [1p36.3]. The absence of the enzyme prevents the remethylation of homocysteine to methionine, and therefore leads to a hyper-homocystinemia and hypo-homocystinuria. Usually, it manifests through early severe neurological signs a apnea, microcephaly and seizures. MTHFR deficiency can also have a late expression while combining Intellectual Disability, ataxia, and schizophrenic like disorders.

Schizophrenic symptoms are often very productive with polymorphic and often visual hallucinations. Several cases are described in the literature (Mattson and Shea, 2003; Roze et al., 2003; Gilbody et al., 2007). The psychotic outburst is usually insidious, but can be dramatically prompted during a post-surgical episode. Roze describes the case of a 16 years old girl, with a dissociative state, having an altered course of though and visual and auditory hallucinations, during 3 months. She used to be a responsible student without major difficulties and did not exhibit any abnormal development trait before the onset of the dissociative state. During the psychiatric interview, only one organic sign was reported: a recent urinary incontinence. Neurological examination revealed an areflective paraparesia and a right plantar extension reflex. Further diagnosis explorations and complete neurological examination were carried out due to familiar history of MTHFR deficiency on the older sister, 24 years old.

Diagnosis is based on amino acids chromatography and the determination of homocysteine. It is then shaped by the sequencing of the gene.

Treatment consists on the association of folate and vitamin B12, or a diet low in methionine/rich in cysteine. Pyroxine can be administered among some patients that reveal to be good responders. These kinds of measures were applied on the 16 years old girl, allowing a significant improvement. Antipsychotic medication was not needed.

## Urea cycle disorders (UCDs)

The urea cycle is the metabolic process allowing nitrogen removal from the body. The lack of one of the six enzymes involved in this cycle interrupts the process and causes the accumulation of nitrogen in the form of hyperammonemia. The prevalence of six pathological conditions related to the urea cycle disorders is 1/8000 births. The intensity of the disturbance is related to the magnitude of the enzyme deficiency. There are milder forms usually involving nausea, vomiting, headache, especially on subjects under high protein diets. Corticosteroids and valproic acid can also worsen the symptoms. Psychiatric signs are not uncommon and may emerge as mood disorders or intense episodic hallucinations (auditory and visual), appearing at acute or subacute periods (Arn et al., 1990; Myers and Shook, 1996; Bachmann, 2003; Enns et al., 2005; Krivitzky et al., 2009; Thurlow et al., 2010). The combination of acute visual hallucinations and vomiting in a context of the introduction of medication or high protein content diet, can lead to suspect an UCD. We have found in the literature a case report of delayed diagnosis due to clinical presentation of anorexia nervosa (Legras et al., 2002).

Diagnosis should be a simple and inexpensive ammonia assay. Main interest on performing an accurate analysis is to introduce a suitable treatment by dietary protein restriction. Some case reports have described mortal consequences of diagnosis delay on psychiatric patients having acute noisy symptoms (Panlaqui et al., 2008).

## Acute porphyria

Porphyrias are a group of eight NMDs causing intermittent neuro-visceral and cutaneous attacks, concomitant or isolated. All porphyrias are generated by enzymes deficit of heme metabolism, leading to an aggregation of porphyrin or its precursors [delta-aminolevulinic acid (ALA) and porphibilinogène (PBG)]. This accumulation is essentially localized in the liver and bone marrow. The prevalence of these disorders is estimated at 5.4/1,000,000. Clinical signs usually appear in adults, although it is not uncommon to find childhood forms. Two porphyria types are known: hepatic or erythropoietic. Both types can encompass chronic courses in the absence of any psychiatric sign. However, acute hepatic forms may manifest by severe abdominal pain, along with nausea, vomiting and constipation, and neurological and psychiatric symptoms.

The diagnosis is based on the measurement of ALA on blood test and PBG in urine. Treatment consists of injection of human hemin and carbohydrates infusion.

The literature on cases of psychiatric events is large and reflects a recurrence of 24–70% over acute patients (Goldberg, 1959; Cashman, 1961; Stein and Tschudy, 1970; Brodie et al., 1977; Bonkowsky and Schady, 1982; Tishler et al., 1985; Boon and Ellis, 1989; Mandoki and Sumner, 1994; Kuhnel et al., 2000; Gross et al., 2002). Frequent clinical founds consist on delusions, psychotic traits as hallucinations, course of thought disorders, and mood disorders (namely depressive type). Some authors even consider that delusion states are as frequent as 40% on patients with acute porphyria (Bonkowsky and Schady, 1982).

A number of cases are particularly illustrative. Santosh and Malhotra (1994) reported a case: 14 years old boy with discrete neurological or organic signs and normal laboratory and imagery results. The patient endured six episodes, more or less severe, and usually escorted by organic minor compounds that were in a way “hidden” by the intensity of psychiatric symptoms. Episodes consisted on a large variety of symptoms, going from disorientation, muteness, echolalia, delusions, hallucinations, hypomania, and even catatonia. Another author describes a similar case in a 50 year old adult (Crimlisk, 1997). However, after a retrospective case review based on the emergency services medical report, it was found recurrent abdominal complains, headache or nausea.

## Wilson disease

Wilson disease is, as for previously described NMDs, an autosomal recessive condition. Even though WD is more common (6/100,000). It is due to the mutation of a gene [ATP7B] encoding for a copper transportation protein, leading to a copper accumulation in the liver, kidney, bones, and brain. The current diagnosis is made on MRI indicating thalamic and lenticular nucleus hyper-signals. Basal ganglia hypo-densities are present in only 25% of subjects. Blood copper measurement and the existence of a conventional Kayser-Fleisher ophthalmic ring, allow easy diagnostic clues. The treatment is based on copper chelation.

Psychiatric symptoms are early symptoms, concerning about two-thirds of all this population (Barthel and Markwardt, 1975; Dening and Berrios, 1990; Schwartz et al., 1993). In general, it has been estimated that one in two patients present psychiatric signs, some authors say one of five, in the absence of any other organic sign (Dening and Berrios, 1989). Three types of psychiatric symptoms are frequently cited on the literature, with a possible simultaneous expression: (i) Mood disorders are the most common sign in terms of psychiatric disorders, including both depressive and manic elements: (Medalia and Scheinberg, 1989; Akil et al., 1991; Dening, 1991; Akil and Brewer, 1995; Srinivas et al., 2008); (Ii) Personality changes are common, particularly with the emergence of irritability and aggressive behavior (Barthel and Markwardt, 1975; Coscia et al., 1975); (Iii) less common, but not less severe, the schizophrenia-like symptoms, usually with catatonic presentation (Barthel and Markwardt, 1975; Renaud et al., 1975; Dening, 1985; Dening and Berrios, 1989; Dening, 1991). An initial report published on Akil and Brewer (1995) founded a 10% frequency of Wilson Disease having schizophrenic-like disorders. A more recent meta-analysis has outlined a 2.4% frequency (Taly et al., 2007).

However, hallucinations are often reported and they are considered, by far, as the more recurrent psychiatric symptom (Sagawa et al., 2003; Wichowicz et al., 2006; Spyridi et al., 2008; Stiller et al., 2008). Early diagnosis is particularly important due to consequences on school and learning. Complex cognitive disorders involving executive functions, memory and neuro-visual., are commonly reported on Wilson's disease patients.

Antipsychotic treatments represent a main frustration. It is often said, in a large number of reports, that antipsychotics exacerbate psychiatric disorders, which can also be a warning sign to make an active search of WD diagnosis. First and foremost, there is an increased risk of developing antipsychotics neurological side effects (Tu, 1981). Second generation antipsychotics are less concerned than first generation ones. Electroconvulsive therapy has proven to be an effective therapeutic alternative, and may be indicated in front of massive antipsychotics side effects or hepatic insult (Shah and Kumar, 1997).

## Niemann pick type C disease (NPC)

The NPC is an autosomal recessive disease associated with a mutation of the NPC1 gene in 95% of cases and NPC2 on 4%. The metabolic mechanism is more complex than for other pathologies mentioned in this article, due to the absence of enzyme deficiency. Indeed, mutations produce a defect in the intracellular transport of cholesterol, glycosphingolipids and sphingosine, leading to their abnormal storage inside the lysosomal compartment. The accumulation is found in various tissues, particularly in the liver and spleen for derived unesterified cholesterol and gangliosides GM2 and GM3 in the brain. Therefore, clinical presentation is extremely diverse with a plenitude of non-specific symptoms.

Early signs can come out before the age of 10 years, mainly neurological. Although, many diagnoses are made on systemic, non-specific isolated signs as neonatal jaundice or splenomegaly. Late diagnoses, sometimes up to 70 years, have very different presentations, and psychiatric signs are not rare. It is estimated that the age of onset of neurological signs is a key element. Usually, non-infantile forms are presented by neurological signs of ataxia type dysarthria, dysphagia, deafness, or cataplexy.

One of the most important signs is supra-nuclear gaze palsy (SNP), constituting an early and almost always presenting sign. Although simple, the SNP is often not explored during medical examination. Eye movements are possible if the examiner asks the patient to follow his finger or a pen, but they become impossible when the subject attempts to do it spontaneously. The diagnosis is made by skin biopsy, Filipin staining test and fibroblast culture. Drug treatment is Miglustat, slowing the progression of the disease (Patterson et al., 2007; Bonnot et al., 2011).

The development of psychiatric symptoms is therefore an exceptional opportunity for early diagnosis and treatment (Vanier, 2010).

NPC cases exhibiting schizophrenia like disorders, without neurological signs, have been reported in the literature. These are not uncommon in clinical practice (for a review see Bonnot et al., 2011). Additionally, there were some interesting cases of schizophrenia with developmental history of autism spectrum-like disorders (Sandu et al., 2009).

In most cases, visual hallucinations are a crucial finding, although this is quite an unusual sign in the classical forms of schizophrenia. Despite the absence of a typical cognitive profile in these patients, the existence of a frontal syndrome is often found, namely through a lack of interpersonal distance. This excessive familiarity can be an early alerting clinical signal (Klarner et al., 2007).

## Cerebrotendinous xanthomatosis (CTX)

The CTX is an autosomal recessive pathology altering the synthesis of bile acid. It is caused by a mutation of the CYP27A1 gene, located on the long arm of chromosome 2. This gene encodes the 27-sterol-hydroxylase involved in the synthesis of cholic and chenodeoxycholic acids. Al altered synthesis of these products, facilitates the accumulation of cholesterol and cholestanol in the brain and tissues, particularly tendons. Xanthomas, consisting on lipids accumulation in the superficial dermis, are a visible core clinical sign. Its prevalence is estimated at 2/100,000 (Bonnot et al., 2010).

The classic clinical presentation is a variable combination of childhood developmental disabilities, juvenile cataracts, chronic diarrhea and epilepsy. Neurological decline and psychiatric symptoms often appear during adolescence. The neurological signs are often a progressive spastic paraparesis, cerebellar ataxia, polyneuropathy, and cognitive impairment. Tendon xanthomas are not rare, constituting a clear distinctive sign. MRI shows a typical dendritic hyper-signal on cerebellar nuclei regions. Diagnosis confirmation is made by plasma cholestanol measures and gene sequencing.

Acute schizophrenic like episodes have been described. Although, other behavioral disorders are largely more frequent amid childhood and adolescence, especially Attention Deficit Hyperactivity Disorders (ADHD) (Sedel et al., 2007). Altogether, psychiatric symptoms concern around 10% of CTX patients, under diverse forms including depression, psychosis, and ADHD (Fraidakis, 2013).

Treatment is based on chenodeoxycholic acid.

## NMDs and psychiatric symptoms overview

Based on collected data and literature review on clinical observations, we propose a comprehensive list of atypicalness key signs and useful warning signs leading to the search for organic pathology, especially a neurometabolic disease.

## Discussion

Schizophrenia spectrum disorders are associated with a wide variety of diseases, both neurological, immunological, neuro-metabolic, genetic, endocrinological, as well as other related conditions induced by medications or toxic substances.

Organic diseases in these cases are called “associated,” while schizophrenia is “secondary.” It is worthy to highlight the fact that organicity in psychiatric conditions makes part of a long date discussion about the origins of mental illness in general (for a historic overview of this discussion, see Conrad, 1960; Neumarker, 2001). Indeed, psychiatric diseases due to organic condition have a distinct recognition DSM or ICD classification, distinguished from to the so called “pure” psychiatric diseases. The IEM described below show that cognitive and symptomatic sequels are common in this field. Moreover, most of the psychiatric symptoms may decrease after treatment of organic condition, especially in disease that affect subjects during development. IEM help us to understand the multidimensional developmental aspects of the psychiatric diseases.

The neurometabolic disorders assemble a large number of diseases, for some of them, unfortunately quite unknown. One of the major interest on reinforcing communication of the reality of these pathologies, comes from the existence of specific medications, some of them new and unfamiliar. The organicity in psychiatry is recognized as an essential element to explore in front of any initial psychiatric sing, still somatic explorations have yet to become a priority on clinical practice. The studies on the prevalence of organic disorders are rare, mainly because they face a major structural methodological difficulty. Undoubtedly, the prevalence of schizophrenic disorders is estimated at 1% of the general population, while that associated pathologies is much lower. Although an exhaustive search is not possible. However, one possible strategy is to approach these data by subpopulation studies. First, studies must be conducted on early forms of schizophrenia related symptoms that has been already reported in association with organic pathologies (Remschmidt and Theisen, 2012). Then, catatonia outburst on childhood and adolescence, mostly associated with schizophrenia-like disorders, for which the largest case series record organic pathologies on 10–15% of cases (Cornic et al., 2009).

Furthermore, it is important to remind the common association between a genetic dimension and schizophrenia, given by the 22q11 micro deletion. This condition is estimated to be present in 1–2% of schizophrenic patients and in 2–4% for the early onset forms (Vorstman et al., 2006; Hoogendoorn et al., 2008). Allegedly it is possible to claim the existence of a significant proportion of patients with schizophrenia related disorders, associated with undiagnosed organic pathologies, although it is difficult to determine their prevalence in an accurate way.

It seems both unrealistic and unnecessary to transform each psychiatrist into an expert on rare diseases in general and neurometabolic disorders in particular. Psychiatry is mainly a clinical discipline, based on observation and the search of characteristic clinical signs in the search of the most suitable diagnosis. There are no idiosyncratic unequivocal signs and a large number of pathologies can share similar clinical features. Semiology is extremely rich and detailed; it is not infrequent to spot some signs that are not usually part of a given clinical picture. We dare to call these clinical signs “atypicalness.”

Most of the atypical signs have been little described, however we dispose of a handful number of studies investigation populations of psychotic patients with associated disorders (schizophrenia and organic pathology without distinction) (Cutting, 1987; Barak et al., 2002; Horiguchi et al., 2009). Heterogeneity of the possible co-morbidities between neurometabolic disorders and psychiatric symptoms could explain, in a sense, the weakness of available data. It seems legitimate to imagine that a genetic or chronic disease can express in diverse manners during an acute episode after particular food exposure or drug intoxication.

Catatonia is considered as an atypical symptom, even when exhibited by elder or younger patients. In the literature, it is often described that visual hallucinations, especially if they are larger than the acoustic-verbal hallucinations classic, are also very suggestive of atypicalness. Antipsychotic treatment resistance is also a frequently found element in some organic conditions. The existence of adverse drug reactions is a key warning point.

Case reports analyses describe a frequent incidence of antipsychotic sides effects, especially neurological. Extrapyramidal effects (akinesia, dystonia, dysarthria, and other akathisia) are particularly frequent in children under antipsychotic treatment (Cohen et al., 2012). Some authors consider that these kinds of side effects could induce a misunderstanding of the clinical presentation and a delay on the accurate detection of underneath neurological signs.

Furthermore, the unusual existence of a rapid onset of psychiatric disorders could be linked to an organic underlying disease. Other listed warning symptoms are the presence of intellectual disabilities, not directly associated to a schizophrenia picture, or a cognitive deterioration in a normal developing child.

Laboratory tests screening for neurometabolic abnormalities is not recommended for all patients, but they are legitimate in case of a high suspicion of neurometabolic disorder.

## Conclusion

An extensive literature review highlights the fact that it is not uncommon to find the existence of hidden organic pathologies, particularly neurometabolic disorders, in association with psychiatric disorders. A large variety of symptoms has been reported, as autism-like symptoms, mood and anxious disorders, as well as behavioral, learning and cognitive disorders. Schizophrenia like symptoms are by far the most frequent psychiatric warning sing of neurometabolic disorders. Thus, it is crucial to make an early detection and diagnosis, especially when they are treatable, as in the case of the seven NMDs presented in the present review.

Purportedly, it will be quite useful to formalize a systematic clinical research in front of psychotic atypical signs and develop easy and usually low-cost measure, having a potential incidence on public health and a massive impact on patients' quality of life.

### Conflict of interest statement

The authors declare that the research was conducted in the absence of any commercial or financial relationships that could be construed as a potential conflict of interest.
